# Efficient Role of Endophytic *Aspergillus terreus* in Biocontrol of *Rhizoctonia solani* Causing Damping-off Disease of *Phaseolus vulgaris* and *Vicia faba*

**DOI:** 10.3390/microorganisms11061487

**Published:** 2023-06-02

**Authors:** Amer M. Abdelaziz, Deiaa A. El-Wakil, Amr H. Hashem, Abdulaziz A. Al-Askar, Hamada AbdElgawad, Mohamed S. Attia

**Affiliations:** 1 Botany and Microbiology Department, Faculty of Science, Al-Azhar University, Cairo 11884, Egypt; drmohamedsalah92@azhar.edu.eg; 2 Department of Seed Pathology Research, Plant Pathology Research Institute, Agricultural Research Center, Giza 12619, Egypt; de107@yahoo.com; 3 Department of Botany and Microbiology, Faculty of Science, King Saud University, Riyadh 11451, Saudi Arabia; aalaskara@ksu.edu.sa; 4 Integrated Molecular Plant Physiology Research (IMPRES), Department of Biology, University of Antwerp, 2610 Antwerp, Belgium; hamada.abdelgawad@uantwerpen.be

**Keywords:** *Rhizoctonia solani*, *Aspergillus terreus*, damping-off, legumes, endophytes, seeds, antifungal activity

## Abstract

The wide spread of plant pathogens affects the whole world, threatening national food security. Various fungi including *Rhizoctonia solani* induce the fungal disease damping-off that negatively affects plant seedlings’ growth. Recently, endophytic fungi are used as safe alternatives to chemical pesticides that harm plant and human health. Here, an endophytic *Aspergillus terreus* was isolated from *Phaseolus vulgaris* seeds to control damping-off diseases by improving the defense system in *Phaseolus vulgaris* and *Vicia faba* seedlings. Endophytic fungus was morphologically and genetically identified *Aspergillus terreus*, and it is deposited in GeneBank under accession OQ338187. *A. terreus* demonstrated antifungal efficacy against *R. solani* with an inhibition zone at 22.0 mm. Moreover, the minimum inhibitory concentrations (MIC) of ethyl acetate extract (EAE) of *A. terreus* were between 0.3125 and 0.625 mg/mL to inhibit *R. solani* growth. Precisely 58.34% of the *Vicia faba* plants survived when *A. terreus* was added compared with the untreated infected (16.67%). Similarly, *Phaseolus vulgaris* achieved 41.67% compared to the infected (8.33%). Both groups of treated infected plants showed reduced oxidative damage (reduced Malondialdehyde and hydrogen peroxide levels) as compared to untreated infected plants. Reduced oxidative damage was correlated with the increase in photosynthetic pigments and the antioxidant defense system including polyphenol oxidase, peroxidase, catalase, and superoxide dismutase enzyme activities. Overall, the endophytic *A. terreus* can be considered an effective tool to control the suppression of *Rhizoctonia solani* in legumes, especially *Phaseolus vulgaris* and *Vicia faba*, as an alternative to synthetic chemical pesticides that harm the environment and human health.

## 1. Introduction

Plants referred to as legumes are regarded as a good source of dietary plant protein. The primary significant economic legume implemented for producing human food and cattle fodder globally is *Vicia faba* (VF). In Egypt, VF is one of the most important economic legume crops as a supplier of protein, carbohydrates, minerals, fat, phosphorus, iron, calcium, and nutritional supplements in food. Furthermore, it plays an ecological role in enhancing N and P nutrition and boosting soil quality [1,2]. Among the most substantial legume crops in Egypt is *Phaseolus vulgaris* (PV), which is grown for domestic and global markets. One of the main sources of protein is PV due to its abundance in the amino acids lysine and tryptophan, which are deficient in grains and other meals [3]. Severe crop losses result from the plant’s exposure to biotic and abiotic stressors. Among the most dangerous plant diseases is a fungal infection which can cause crops to entirely or partially fail and reduce crop quality, endangering the safety of the world’s food supply [4,5]. There are numerous methods of managing plant diseases, including chemically through synthetic pesticides, biologically using beneficial microorganisms, agriculturally using processes, and genetically [6]. *Rhizoctonia solani* is a fungus that inhabits soil but is very destructive to plants [7,8]. Endophytic fungi are an excellent supplier of a variety of biologically active substances that are employed in both the medical and agricultural fields of the economy in order to treat disease [9,10]. Endophytic fungi can defend plants from diseases through colonization sites, nutritional competition with pathogens, antibiotic synthesis, and induction of resistance mechanisms [11]. The endophytic fungi may help the host plant grow by producing phytohormones or enhancing the plant’s resistance to different stresses and can synthesize poisons to protect plants from herbivores [12,13,14,15]. Plants have the ability to generate enormous pools of interconnected microorganisms, such as bacteria and fungi that are actinomycetes which are known as plant micro biomes [16]. Endophytic fungi are considered a strong source of secondary metabolites [17]. Endophytic fungi are a very important and hyper-diverse type of endophytes thought to include up to one million distinct fungal taxa [18,19]. One of the most prevalent and common fungal endophytes is the *Aspergillus* fungus. *Aspergillus* extensively applies fungi in the struggle versus biotic and abiotic plant diseases. Furthermore, *Aspergilli* serves as a plant growth stimulant through nitrogen fixation, phosphate solubilization, indole acetic acid production, and hydrogen cyanide production [20,21,22]. Herein, this study aimed to isolate and identify an effective endophytic fungus as in vitro antifungal agents against *R. solani* to biocontrol the damping-off disease of *Phaseolus vulgaris* and *Vicia faba*.

## 2. Materials and Methods

### 2.1. Sampling Process

Samples of common bean (*Phaseolus vulgaris* L.) cv. Nebraska seeds were obtained from the Vegetable Research Department, Horticulture Research Institute, Agricultural Research Center, Egypt. The obtained seed samples were divided into two working samples and then were put in paper bags and numbered; after this, they were preserved at 4 °C for further studies.

### 2.2. Isolation and Identification of the Endophytic Fungi

Fungal endophytes were isolated from common bean (*Phaseolus vulgaris* L.) cv. Nebraska seeds according to the method used by Abdelaziz et al. [15]. Two hundred healthy-looking seeds of common beans were washed with sterilized distilled water twice and then sterilized with 70% ethanol for one minute and with 4% NaOCl for one minute. Seeds were plated in sterile Petri dishes (10 cm diameter) at the rate of 10 seeds per each Petri dish. The plates were kept in the incubator under dark conditions for one week at 25 ± 2 °C and monitored daily. Then, the growing mycelium was picked carefully, sub-cultured, and purified by the single-spore technique. The isolated, purified fungi were screened according to their antifungal activity against *R. solani*, and the most potent fungus was identified morphologically based on the variation in colonies, morphological characters, and genetics according to Attia, Hashem [23]. Finally, the obtained isolates were kept in a refrigerator at 4 °C for further studies.

### 2.3. Source of the Pathogenic fungus and Inoculum Preparation

*Rhizoctonia solani* RCMB 031001 was obtained from the Regional Center for Mycology and Biotechnology (RCMB), Al-Azhar University, Cairo, Egypt, and then it was confirmed by the pathogenicity test according to El-Batal et al. [24]. The isolate was grown on potato dextrose agar medium (PDA) plates for 7 days at 28 ± 2 °C before being stored at 4 °C. The pathogenic fungus *Rhizoctonia solani* inoculum was prepared according to Hashem et al. [25].

### 2.4. GC-MS Study of Fungal Metabolites Extracted from Ethyl Acetate

Endophytic *A. terreus* was grown for 14 days on potato dextrose broth (PDB) at 28 ± 2 °C under static circumstances. Afterward, the culture was filtered through Whatman No. 1 filter paper. The resulting culture filtrate was mixed with ethyl acetate in a 1:1 ratio and collected in the uppermost portion of the organic layer. Furthermore, the extraction method was repeated three to four times, pooled, and condensed at 45 °C using a rotary evaporator. After that, the ethyl acetate extract (EAE) was collected and stored at room temperature [21]. To determine the metabolic components, the EAE of *A. terreus* was put into a GC-MS. The GC-MS analysis was carried out using an Agilent Technologies GC-MS 5977 A at 70 eV using a computer mass spectral library (NIST, 2011 edition). The unknown components’ spectra matched the data in the GC-MS library.

### 2.5. In Vitro Antifungal Activity of EAE of Endophytic A. terreus

The antifungal activity of EAE of endophytic *A. terreus* was examined using the well diffusion method. *R. solani* was plated on PDA and incubated at 28 ± 2 °C for 3 to 5 days after inoculation. On the sterile and solidified PDA medium, *R. solani* inoculum was evenly distributed. Wells (7 mm) were cut using a sterile cork borer; 100 µL of EAE (5 mg/mL) was transferred to each well individually and left for 2 h at 4 °C. After incubating the plate at 28 ± 2 °C for 5 days, the inhibition zones were observed and measured. Moreover, minimum inhibitory concentration (MIC) was carried out using agar well diffusion method; different concentrations of EAE (5, 2.5, 1.25, 0.625, and 0.3125 µg/mL) were separately prepared in tubes; then 5 wells (7 mm) were made on inoculated PDA medium with *R. solani*; and 100 µL of each concentration was put in the well, respectively, and incubated at 28 ± 2 °C for 5 days. Then, the inhibition zone for each concentration was observed and measured [23].

### 2.6. In Vivo Assessment Activity of A. terreus on V. faba and P. vulgaris L.

The faba bean (*Vicia faba*) cv. Giza 3 (VF) and common bean (*Phaseolus vulgaris* L.) cv. Nebraska (PV) were obtained from the Agricultural Research Center, Egypt. Seeds of equal sizes were surface sterilized by submerging them for 2 min in 2% NaOCl and then washed with distilled, sterilized water. The treatments were designed (three replicates for each treatment) as follows: T1, control healthy: The sterilized VF or common bean seeds were submerged in sterilized distilled water for three hours. T2, control infected: The sterilized VF or common bean seeds were submerged in sterilized distilled water inoculated with *R. solani* for three hours. T3: The sterilized VF or common bean seeds submerged in sterilized distilled water were inoculated with endophytic AT for three hours. T4: The sterilized VF or common bean seeds were submerged in sterilized distilled water inoculated with *R. solani* and endophytic AT for three hours. All treatments were allowed to germinate in plastic trays. Disinfected sand and clay were mixed in a 1:3 ratio for the plastic trays used for seed germination and kept in the greenhouse at a temperature of 22 °C during the day and 18 °C at night, with a relative humidity of 70–85%. The seeds were irrigated routinely with 24 seeds per treatment according to Khattab et al. [26]. Pre-emergence damping-off was measured after 15 days, while post-emergence damping-off and survival were measured after 30 days according to Hashem et al. [25].

### 2.7. Biochemical Indicators

To find the pigment content, fresh 0.5 g leaf tissue was crushed in acetone (80%). The filtrate was centrifuged for five minutes at 10,000 rpm, and the filtrate’s absorbance was measured at 665, 470 and 652 nm to calculate the carotenoid, chlorophyll a, and chlorophyll b contents [27]. To calculate the phenolic content, 5–10 mL of ethanol (80%) was used to extract 1 g of plant tissue for 24 h, and the residue was twice extracted with the same solvent after filtration. With 80% ethanol, all extracts have been completed to a total volume of 50 mL. Folin’s reagent (0.5 mL) and the extract (0.5 mL) were well combined before shaking vigorously for three minutes. A saturated Na_2_CO_3_ solution (1 mL) was added, followed by thoroughly homogenizing it with distilled water (3 mL). A spectrophotometer was used to measure the developed blue color at 725 nm after one hour [27].

For free proline determination, homogenization of 0.5 g of dried plant material with 10 mL of sulfo-salicylic acid (3%) was performed. In a reaction involving 2 mL of filtrate, 2 mL of acid ninhydrin, and 2 mL of glacial acetic acid, the filtrate was subjected to filtration. After one hour in a bath of boiling water, this reaction was placed in a cold bath for one hour. Next, 4 mL of toluene was utilized to extract the reaction mixture. Then, the color obtained was measured at 520 nm [28]. The technique of Hu, Richter [29] was applied to analyze the MDA content in the fresh plant leaves as follows: Fresh leaf samples were centrifuged at 4000 rpm for 10 min after being extracted with 5% trichloroacetic acid. A 0.6% Thiobarbituric Acid solution was added to 2 mL of the leaf extract, and the combination was then immersed in a water bath for 10 min. After cooling, the color’s decreasing absorbance was at 532, 600, and then 450 nm. Malondialdehyde concentration was determined using the formula 6.45 (A532–A600)/0.56 A450.

Fresh leaves also were recognized for hydrogen peroxide H_2_O_2_. For extraction, 4 mL of acetone was combined with 0.5 g of fresh leaves. After that, 3 mL of the mixture was combined with 1% titanium dioxide that had been dissolved in 20% H_2_SO_4_ before being centrifuged at 6000 rpm for 15 min. Then, at 415 nm, the developed yellow color was measured [30]. The enzyme extract was made in the manner described below. First, 14 days after seed germination, 1 g of the terminal buds and the first and second young leaves were homogenized with 5 mL of phosphate buffer pH 6.8 and centrifuged at 2 °C for 20 min at 20,000 rpm, and the enzyme activity was assessed in the clear supernatant. The accepted method of Srivastava, ref. [31] was used to determine peroxidase (POD). The solution used to determine POD contained 5.8 mL of 50 mM phosphate buffer pH 7, 0.2 mL of enzyme extract, and 2 mL of 20 mM H_2_O_2_ after being added to 2 mL of 20 mM pyrogallol. Within 60 s at 470 nm and 25 °C, the UV spectrophotometer (Jenway) was used to spectrophotometrically quantify the rate of rise in absorbance as pyrogallol. The activity of the polyphenol oxidase (PPO) enzyme was splendid using the technique of Matta [32]. For the purpose of determining PPO, a 125 µmol solution of phosphate buffer (pH 6.8), 100 µmol of pyrogallol, and 2 mL of enzyme extract were utilized. After five minutes at 25 °C of incubation, the reaction was halted by adding one milliliter of 5% H_2_SO_4_. The enzyme extract used to create the blank sample was very thoroughly boiled, and the generated color was detected at 430 nm. Catalase (CAT) activity was assayed by monitoring the change in absorbance at 240 nm for 2 min in 1 mL reaction mixture that contained 100 mM phosphate buffer (pH 7.0), EDTA, H_2_O_2_, and 100 µL enzyme extract [33]. Marklund and Marklund’s technique [34] for measuring superoxide dismutase (SOD) activity was used. The solution (10 mL), which was used to determine the amount of SOD, contained 3.6 mL of pure water, 0.1 mL of enzyme, 5.5 mL of 50 mM phosphate buffer (pH 7.8), and 0.8 mL of 3 mM pyrogallol (dissolved in 10 mM HCl).

### 2.8. Statistical Analysis

One-way analysis of variance (ANOVA) was used to analyze experimental data, and the Duncan’s multiple range test and the (LSD) at a probability level of 5.0% were used to separate means differences.

## 3. Results and Discussion

### 3.1. Isolation and Identification of Endophytic Fungi

**Figure 1:**
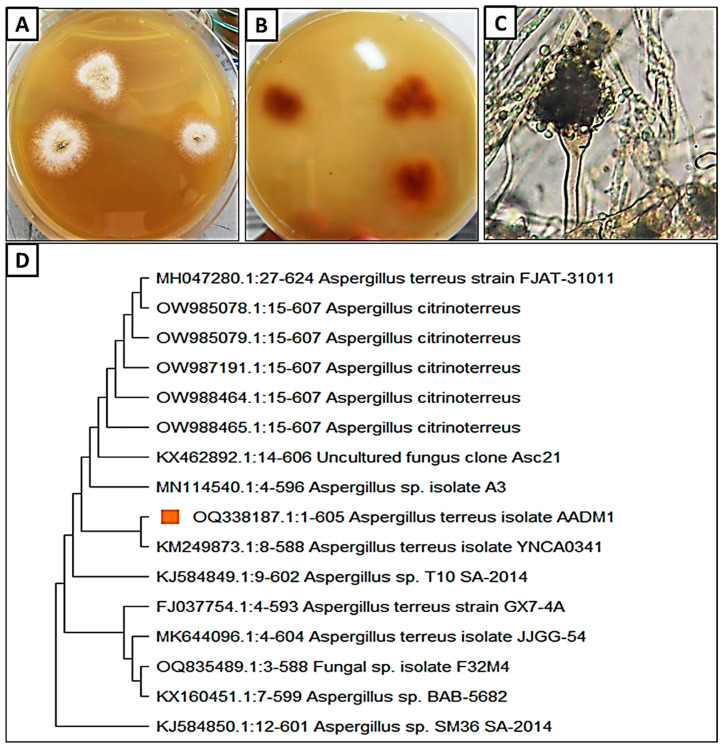
Morphological and molecular identification of *A. terreus* (**A**–**D**): (**A**) Colony of *A. terreus* on PDA; (**B**) Reverse colony, (**C**) Light microscope (400×); (**D**) Phylogenetic tree of the investigated fungus *Aspergillus terreus*.

Seven endophytic fungal isolates (AADM1-AADM7) were isolated from common bean seeds and then screened according to their antifungal activity. Fungal isolate AADM1 was the most potent according to the preliminary screening and identified morphologically and genetically (Figure S1). Morphological identification demonstrated the fungus’s low growth rate with the formation of finely granular conidia at 27 ± 2 °C on PDA medium after seven days, with 15–20 mm in diameter (Figure 1A). The surface is buff, containing reverse yellow to orange pigments that are darker in the middle (Figure 1B), and is enclosed by yellow Hull cells. Mycelium is hyaline and septate, whereas conidiophores are non-septate with smooth-walled; globular vesicles varying in size from 70 to 300 μm are observed as well as 8–12 mm-long cylindrical vesicles with smooth, globose, very small conidia (2–2.5 μm) (Figure 1C). This result was confirmed by Khalil, Ahmed [35]. Furthermore, molecular identification using the ITS rRNA region was used to identify the fungal isolate AADM1 genetically. The obtained sequence was deposited in the Gene Bank under the accession number OQ338187. As shown in Figure 1D, the phylogenetic tree revealed that this sequence was closely linked to the *Aspergillus terreus* sequence and shared 99% identity with it.

### 3.2. Analysis of Crude EAE of Endophytic A. terreus Using GC-MS

**Figure 2:**
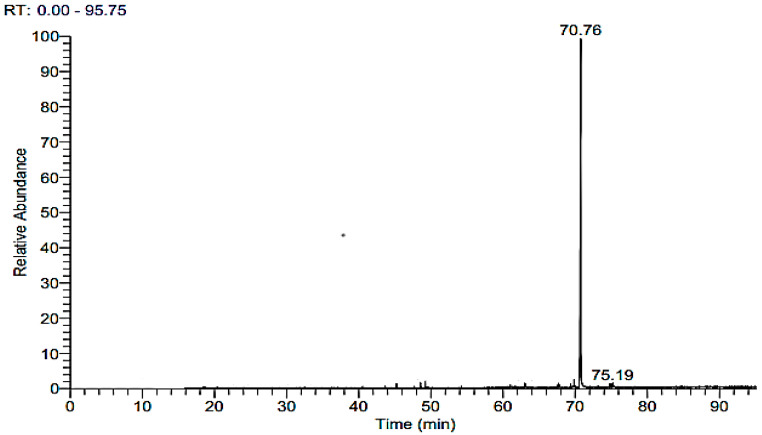
GC-MS chromatogram of crude EAE of endophytic *A. terreus*.

One of the most extensively used methods for assessing phytochemical substances of natural origin [36] is gas chromatography coupled with a mass spectrometer due to its stability, sensitivity, and high efficiency. Antimicrobial, antioxidant, anticancer, and antiviral activity are only some of the many biological effects attributed to bioactive chemicals found in fungal endophytes. Endophytic fungi can produce various antimicrobial secondary metabolites [9,37], including steroids, flavonoids, terpenoids, peptides, quinones, lignans, alkaloids, phenylpropanoids, phenolics, and isocoumarins [23,37]. The results of GC-MS analysis of EAE of *A. terreus* are shown in Table 1 and Figure 2. The results revealed that the EAE of *A. terreus* contains seven different compounds, where the dominant compound was Bis(2-ethylhexyl) phthalate with a ratio of 80.87%. Lotfy, Hassan [38] reported that Di-(2-ethylhexyl) Phthalate was the major bioactive metabolite isolated from *Aspergillus awamori*, where results illustrated antimicrobial activity against the gram-positive bacteria *Sarcina lutea* and unicellular fungi (*Candida albicans*); furthermore, it exhibited anticancer activity against some carcinoma cell lines. Moreover, Javed, Salman [39] isolated Bis-(2-Ethylhexyl) Phthalate from the lactic acid bacteria *Lactiplantibacillus plantarum* in which there appeared antibacterial activity against gram-negative (*E. coli*) and gram-positive bacteria (S. *aureus*). Furthermore, Habib and Karim [40] reported that Di-(2-ethylhexyl) Phthalate, which was isolated from *Calotropis gigantea* (Linn.) flower, has antimicrobial and cytotoxic activity. Furthermore, biologically active minor compounds were Pyrrolo [1,2-a] pyrazine-1,4-dione, 1-Docosene, Palmitic Acid TMS derivative, Stearic acid TMS derivative with ratios 4.49, 1.38, 2.31, and 0.89%, respectively, which were reported previously with antimicrobial and antioxidant activity as shown in Table 1. On the other hand, tow compounds 1-(2,4-Dichloro-phenyl)-N’-hydroxy-cyclopropane carboxamidine and octadecanoic acid, 2,3-Bis [(trimethylsilyl)oxy] propyl ester were detected in the crude extract with 0.70 and 0.75% with no activity according to the previous literature.

### 3.3. In Vitro Antifungal Activity of EAE of Endophytic A. terreus against the Pathogenic R. solani Isolate

**Figure 3:**
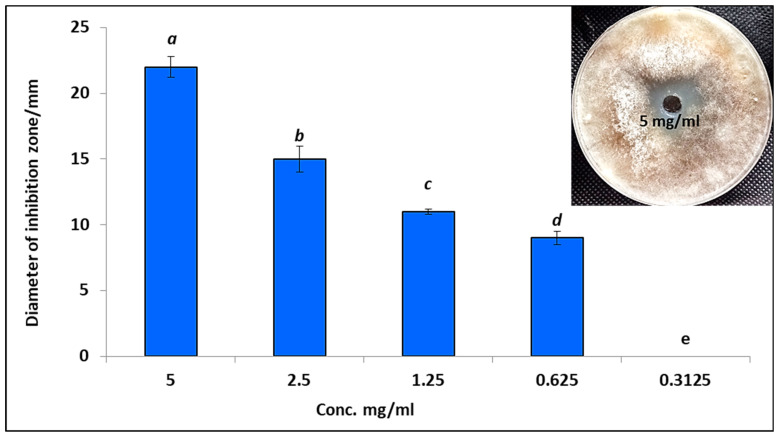
Antifungal activity of crude EAE of *A. terreus* at different concentrations against *R. solani*. Letters a, b, c, d mean power significance. (a, b, c, d and e: symbols of significance letters).

Endophytic fungi are capable of producing a vast array of chemically distinct secondary metabolites [45], which have many functions, including their use as antimicrobials, antifungals, and antivirals [23,46]. In the current study, EAE of endophytic *A. terreus* was assessed as an antifungal agent against *R. solani* using the agar well diffusion method as shown in Figure 3. Results showed that EAE of endophytic *A. terreus* displayed potential antifungal activity toward *R. solani* where the inhibition zone was 22 mm at concentration 5 mg/mL (Figure 3). Furthermore, EAE of endophytic *A. terreus* at different concentrations 2.5, 1.25, 0.625, and 0.3125 mg/mL were evaluated for antifungal activity, where inhibition zones were 15, 11, 9, and 0 mm, respectively. Therefore, the MIC of EAE of endophytic *A. terreus* to inhibit the growth of *R. solani* was between 0.3125 and 0.625 mg/mL.

A prior study isolated *A. terreus* from various plants that have antibacterial and antifungal activity [47]. Another study used endophytic *A. terreus* fungus for biocontrol of sesame wilt disease, pythium-induced damping-off of cucumber [48], and okra-rot-causing Cochliobolus spicifer-CSN-20 [49].

### 3.4. In Vivo Assessment Activity of A. terreus on Vicia faba and P. vulgaris

**Figure 4:**
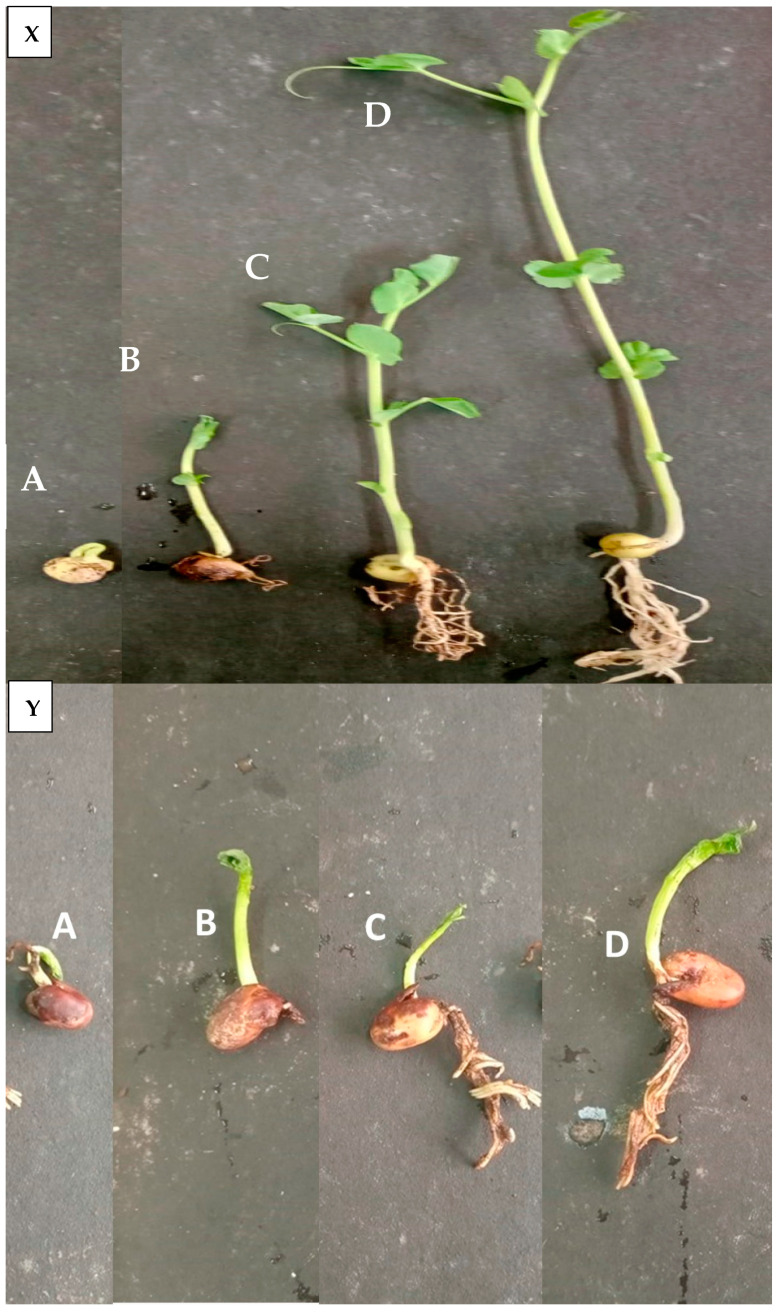
Effect of endophytic *A. terreus* on infected (X) *Phaseolus vulgaris* and (Y) *Vicia faba* seedlings with *R. solani* causing damping-off disease. (**A**) Infected treated with *R. solani* (control); (**B**) Infected treated with *A. terreus*; (**C**) Healthy untreated (control); (**D**) Healthy treated with *A. terreus*.

The results in Table 2 and Figure 4 show that the fungus *R. solani* was extremely aggressive on both VF and PV plants. The outcomes additionally showed that the fungus *R. solani* on the PV plant seeds had higher virulence, rating 91.67% pre- and post-emergent damping-off compared to the VF plant seeds’ 83.33%. The earlier studies’ conclusions of *R. solani* had a strong virulence on PV and VF [50].

The results in Table 2 demonstrate that the fungus *R. solani* severely reduced the germination rate for both VF and PV plant seeds. The inhibition was considerably more severe in PV than in VF. These outcomes are in line with earlier research, which revealed that fungal plant pathogens including *R. solani* greatly slows down seed germination in VF and PV [51,52].

Nevertheless, compared to the infected control, this study proved a beneficial effect of using the endophytic fungus *A. terreus*, resulting in significant improvement in the germination rate. Previous studies suggest that endophytic fungi, mainly Aspergilli, release metabolites that are nutrient-rich for seedlings and may encourage the rate of seed germination [46,53].

### 3.5. Photosynthetic Pigments

**Figure 5:**
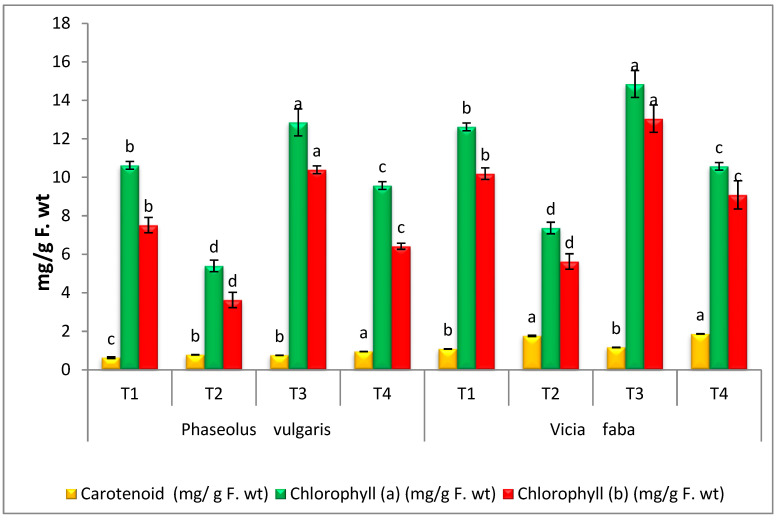
Effect of *A. terreus* on photosynthetic pigments of *R. solani*-infected *P. vulgaris* and *V. faba* seedlings. T1 = Healthy control; T2 = Infected control; T3 = Healthy with *A. terreus*, and T4 = Infected with *A. terreus*. Data are presented as means ± SD (n = 3). Data are from the LSD test at *p* ≤ 0.05. (a, b, c and d: symbols of significance letters).

Photosynthetic pigments (Chl a and Chl b) were highly inhibited in *R. solani* infected PV by 49.1% and 51.7% and VF by 41.6% and 44.7%, respectively. On the other hand, the present study showed that the level of carotenoids in infected PV and VF seedlings increased by 23.4% and 62.58%, compared to the healthy control. However, when healthy seedlings PV and VF were treated with AT, a promising improvement response in comparison to healthy control plants was observed. Moreover, infected PV seedlings treated with AT successfully recovered the loss of Chl. a by 77.22% and chlorophyll b by 76.8%, compared with infected control (Figure 5). Moreover, results indicated that infected VF treated with AT effectively improved the loss of Chl. a by 43.41% and chlorophyll b by 61.45%, compared with infected control. In seedlings infected and treated with AT, contents of carotenoids were increased when being compared with those only infected. Damping-off is a soil-borne fungal disease that destructively impacts the crops, mainly affecting seeds [54]. Damping-off refers to the rotting of stem and root tissues at and below the soil line [55]. The harmful effect of *R. solani* in PV and VF seedlings can be shown to reduce the synthesis of chlorophyll pigments. This study exhibited a retarded photosynthetic pigment in PV and VF seedlings in response to *R. solani*. In this regard, the reduction in chlorophyll may be correlated with different factors—among them are the seedling failure to catch the light and a disorder in growth hormones [56]. The decline in photosynthetic pigments may be due to a deficit in light capture or may also be due to the degradation of chlorophyll by raising the activity of chlorophyll-reducing enzymes and chlorophyllase [5,57]. Reports on the beneficial effects of endophytic fungi on plants are many and supportive of the current study [58,59,60]. The application of AS successfully recovered the loss of Chl. a by 77.22% and chlorophyll b by 76.8%, compared with infected plants. Synthesis of chlorophyll can increase the assembly of energy and perform as a source for essential cellular roles. Similar to Attia et al. [23], these results established that endophytic fungi AT can increase the plant performance and photosynthetic capacity.

### 3.6. Total Phenol and Free Proline Content

**Figure 6:**
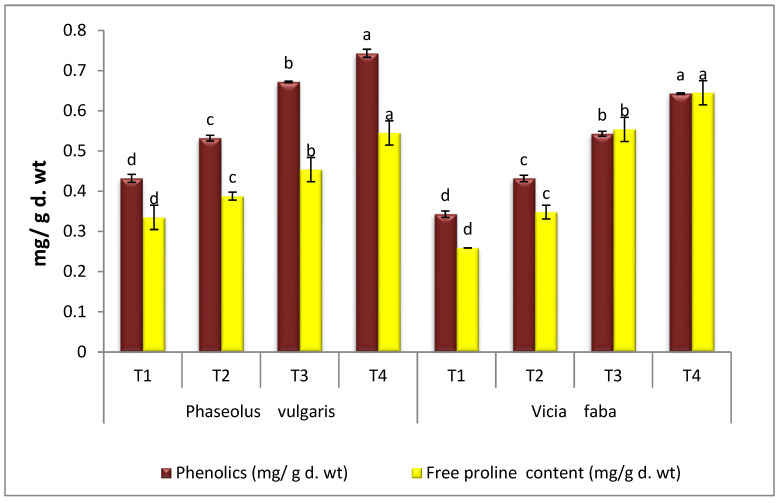
Effect of *A. terreus* on phenolics and free proline content of *Rhizoctonia solani*-infected *P. vulgaris* and *V. faba* plants. T1 = Healthy control; T2 = Infected control; T3 = Healthy with *A. terreus*, and T4 = Infected with *A. terreus*. Data are presented as means ± SD (n = 3). Data are from the LSD test at *p* ≤ 0.05. (a, b, c and d: symbols of significance letters).

Both PV and VF seedlings grown under *R. solani* infection conditions showed significant increases in their contents of total phenols and free proline when compared to the controls. Moreover, the application of AT on infected PV seedlings successfully improved total phenols by 39.69% and free proline by 40.46%, compared with infected control (Figure 6). Furthermore, the results indicated that the infected VF plants treated with AT effectively increased total phenols by 48.84% and free proline by 85.34%, compared with the infected control plants. However, when healthy seedlings PV and VF were treated with AT, a promising improvement of total phenols and free proline in comparison to healthy control plants was observed.

The results in the current study show that proline contents in RS-infected PV and VF seedlings were raised. Similarly, it has been reported that free proline contents were significantly increased in PV and VF when infected with RS, the causative agent of damping-off disease [50,61,62,63]. Regarding the application of AT endophyte, earlier studies have recorded that AT endophyte stimulates the accumulation of proline which is responsible for the defense against the pathogen [23,64,65]. Infected plants treated with fungal endophytes have high levels of proline compared to non-treated plants [23,66,67]. Non-enzymatic pathways include phenolic compounds, which have the ability to affect ROS production [68]. In this study, both PV and VF seedlings infected with RS showed significant increases in the contents of total phenols. These results are suggested also by [25]. The provocation of the pathogen to the plant, when the invasion occurs, leads to an increase in the concentration of phenols and phenol oxidation products, which play an important role in stopping or limiting the progression of the pathogen [69,70,71]. Moreover, the application of AT on infected PV and VF seedlings successfully improved total phenols. However, when healthy PV and VF plants were treated with AT, a promising improvement of total phenols and free proline was observed compared to healthy control plants. Treating the plant with elicitors induces the formation of phenolic compounds that play a role in the induced resistance; the formation of lignin and salicylate; and, finally, stopping or limiting the progression of the pathogen [72]. Numerous reports [23,46] showed an increase in phenols in infected plants after being treated with endophyte AT, which improved plant immunity and reduced infection symptoms.

### 3.7. Oxidative Stress

**Figure 7:**
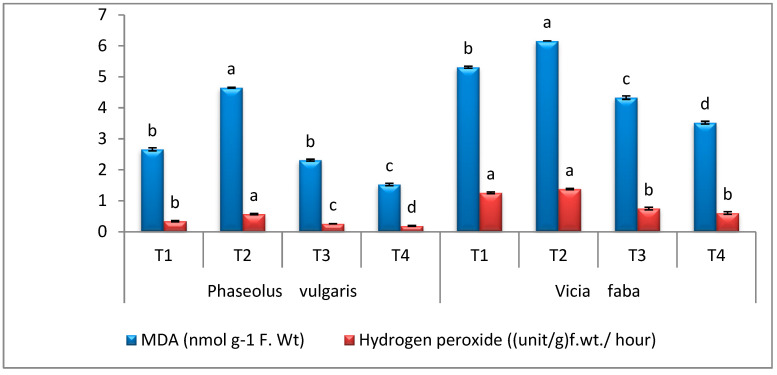
Effect of *A. terreus* on the contents of MDA and H_2_O_2_ of *Rhizoctonia solani*-infected (*P. vulgaris* & *V. faba*) plants. T1 = Healthy control; T2 = Infected control; T3 = Healthy with *A. terreus*, and T4 = Infected with *A. terreus*. Data are presented as means ± SD (n = 3). Data are from the LSD test at *p* ≤ 0.05. (a, b, c and d: symbols of significance letters).

The *R. solani* infection accumulated the contents of MDA and H_2_O_2_ in PV by 50.32% and 54.38%, respectively, compared to the healthy control (Figure 6). Moreover, the application of AT on infected PV plants successfully declined the MDA content by 42.97% and H_2_O_2_ by 40.35% compared to the infected control (Figure 7). The results showed that the RS infection caused an increase in the contents of MDA and H_2_O_2_ in VF by 50.32% and 45.65%, respectively, compared to the healthy control plants. Moreover, the application of AT on infected PV plants successfully declined MDA content by 29.70% and H_2_O_2_ by 8.69% compared to the infected control. However, when healthy PV and VF plants were treated with AT, a promising decline in the contents of MDA and H_2_O_2_ in comparison to healthy control plants was observed. Oxidative stress caused by RS led to a serious disturbance to plant cell and rise contents of malondialdehyde (MDA) and hydrogen peroxide (H_2_O_2_) in PV and VF seedlings. These findings are in harmony with Dikilitas et al. [73]. Results in the current study show that the application of AT on infected PV and VF seedlings successfully declined MDA and H_2_O_2_ compared to the infected control plants. However, when healthy PV and VF plants were treated with AT, a promising decline in the contents of MDA and H_2_O_2_ in comparison to healthy control plants was observed. The application of AT endophyte reduced malondialdehyde and hydrogen peroxide during a rise in antioxidant compounds that scavenge (ROS) and inhibit cellular membranes from oxidative stress [14].

### 3.8. Oxidative Enzymes Activity

**Figure 8:**
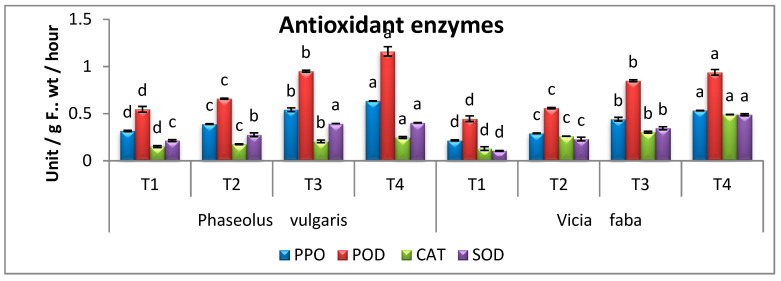
Effect of *A. terreus* on the activities of polyphenol oxidase (PPO), peroxidase (POD), catalase (CAT), and superoxide dismutase (SOD) of *Rhizoctonia solani*-infected *P. vulgaris* and *V. faba* seedlings. T1 = Healthy control; T2 = Infected control; T3 = Healthy with *A. terreus*, and T4 = Infected with *A. terreus*. Data are presented as means ± SD (n = 3). Data are from the LSD test at *p* ≤ 0.05. (a, b, c and d: symbols of significance letters).

To achieve a stronger indication of several defense-responsible enzymes, the mean activities of peroxidase, polyphenol oxidase, superoxide dismutase, and catalase of the tested PV and VF seedlings were clarified in this experiment. Both PV and VF seedlings infected with *R. solani* showed highly significant increases in PPO, POD, CAT, and SOD activities, compared to healthy controls (Figure 8). Moreover, POD, PPO, SOD, and CAT activities were higher in the RS-infected seedlings treated with AT. The application of AT on infected PV and VF plants increased the activity of the antioxidant enzyme PPO by 17.19% and 21.08%, POD by 22.14% and 10.35%, CAT 19.90% and 61.91%, and SOD by 2.27% and 41.62%, respectively, when compared with untreated infected plants (Figure 7). Moreover, when healthy PV and VF seedlings were treated with AT, a promising increase in the activity of antioxidant enzymes (PPO, POD, CAT, and SOD) in comparison to the healthy controls was observed.

Polyphenol oxidase (PPO), peroxidase (POD), catalase (CAT), and superoxide dismutase (SOD) supply a great number of defensive enzymes and are correlated with fungal disease [74,75,76]. These enzymes act as early stages in accumulative resistance to numerous stresses as well as the formation of phenolic compounds [77,78]. Our results indicated that *R. solani* showed highly significant increases in PPO, POD, CAT, and SOD activities in PV and VF seedlings compared to healthy control plants. These results show that a variety of proteins function as scavengers for these ROSs including catalase (CAT) and peroxidase (POD) [79]. The application of AT endophyte was reported to increase the activity of catalase and peroxidase in tomatoes [23], PV [80], and VF [81].

## 4. Conclusions

In the current study, endophytic *A. terreus* was isolated from common bean seeds and identified morphologically and genetically. In vitro, an ethyl acetate extract of endophytic *A. terreus* had potential antifungal activity towards the pathogen *R. solani,* damping-off disease. In vivo, endophytic *A. terreus* was used to improve the systemic resistance of *P. vulgaris* and *V. faba* plants against damping-off disease. As a result, *A. terreus* was used as a bio stimulator to induce the organic compounds responsible for defense, such as proline, phenols, and antioxidant enzymes, against disease. The results showed a significant improvement in the seedlings’ content of chlorophyll, free proline, phenols, and antioxidant enzymes, as well as a reduction in MDA and H_2_O_2_. Thus, EAE of endophytic *A. terreus* can be used as a safe, promising, and eco-friendly bio-fungicide alternative to chemical pesticides for the biocontrol of damping-off disease caused by a *R. solani* infection in two-legume crops.

## Figures and Tables

**Table 1:** GC-MS analysis of crude EAE of endophytic *A. terreus*.

C	Compound	Rt (min)	Peak Area %	Activity	References
1	Pyrrolo [1,2-a] pyrazine-1,4-dion e	49.21	4.49	Antibacterial and antioxidant activity	[41]
2	1-Docosene	54.22	1.38	Antimicrobial activity	[42]
3	1-(2,4-Dichloro-phenyl)-N′-hy proxy-cyclopropanecarboxami dine	67.75	0.70	No activity reported	-
4	Palmitic Acid, TMS derivative	69.84	2.31	Antioxidant activity	[43]
5	Bis(2-ethylhexyl) phthalate	70.75	80.87	Antibacterial, antifungal and cytotoxic activity	[38,39,40]
6	octadecanoic acid, 2,3-Bis [(trimethylsilyl)oxy] propyl ester	74.83	0.75	No activity reported	-
7	Stearic acid, TMS derivative	75.19	0.89	Antimicrobial activity	[44]

**Table 2:** Effect of *A. terreus* on the *R. solani* pre- and post-emergence damping-off.

Treatment		Pre-Emergence Damping off %	Post-Emergence Damping off %	Survival Plant %
***V. faba***	Healthy control	0.0	0.0	100 ^a^
	Infected control	62.5 ^b^	20.83 ^b^	16.67 ^d^
	Healthy + *A. terreus*	0.0	0.0	100 ^a^
	Infected + *A. terreus*	33.33 ^d^	8.33 ^c^	58.34 ^b^
***P. vulgaris***	Healthy control	0.0	0.0	100
	Infected control	66.67 ^a^	25 ^a^	8.33 ^e^
	Healthy + *A. terreus*	0.0	0.0	100 ^a^
	Infected + *A. terreus*	37.5 ^c^	20.83 ^b^	41.67 ^c^

Symbols of significance letters: (a, b, c, d and e).

## Data Availability

Not applicable.

## Supplementary Materials

The following supporting information can be downloaded at: https://www.mdpi.com/article/10.3390/microorganisms11061487/s1, Figure S1: Schematic diagram showing isolation of seed-borne endophytic *Aspergillus terreus*, antifungal activity and plant growth promoting induced by treatment with *Aspergillus terreus*.
