# Supplementary material for: Efficient Role of Endophytic Aspergillus terreus in Biocontrol of Rhizoctonia solani Causing Damping-off Disease of Phaseolus vulgaris and Vicia faba
Source: Microorganisms. 2023 Jun 2;11(6):1487. doi: 10.3390/microorganisms11061487 (PMC10303203; doi:10.3390/microorganisms11061487)
Supplement: Supplementary file 1 [file microorganisms-11-01487-s001.zip › microorganisms-2392362-supplementary.pdf]

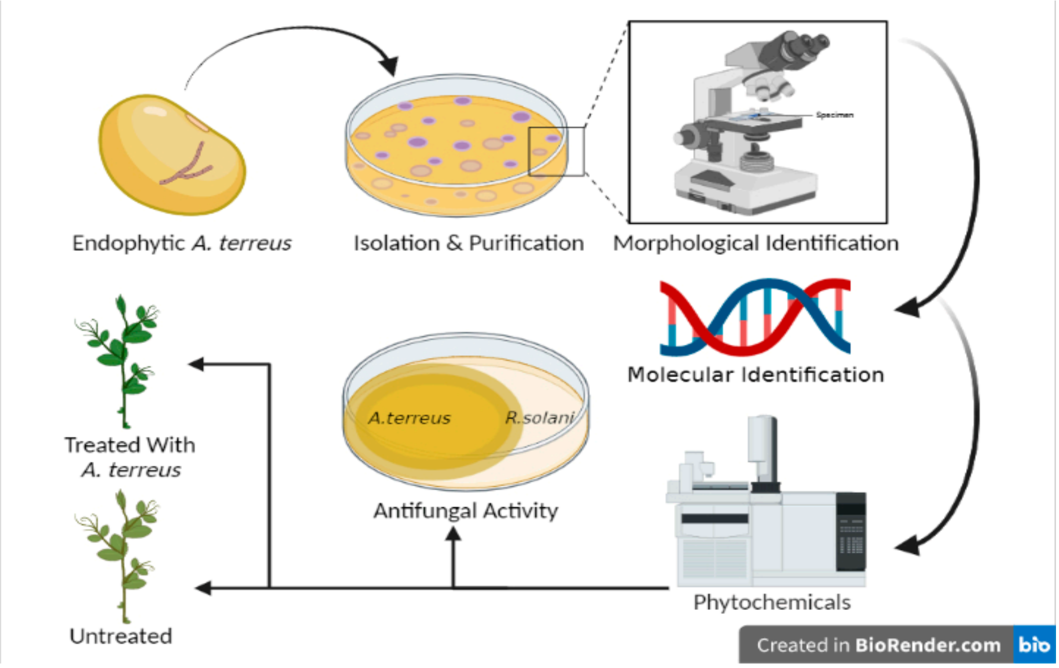

Figure S1. Schematic diagram showing isolation of seed-borne endophytic *Aspergillus terreus*, antifungal activity and plant growth promoting induced by treatment with *Aspergillus terreus*.
